# What’s in a @name?

**DOI:** 10.1038/s44319-024-00194-7

**Published:** 2024-07-01

**Authors:** Howy Jacobs

**Affiliations:** Tampere University, Tampere, Finland

**Keywords:** Careers, Science Policy & Publishing

## Abstract

Can we simplify the way we cite our names, affiliations and email addresses in our academic production?

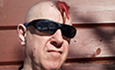

Everyone involved in scientific research is concerned that they should be duly credited for their work and contributions, usually in the form of publications. This applies both to the individuals who conduct the research as well as to the institutions that host it.

For individuals, we at least have the ORCID system to ensure that those who change their name, gender or affiliation, or whose name is common, variably spelled—such as with or without diacritic marks—or liable to being permuted or otherwise misconstrued, are given due recognition for what they have accomplished. Although it’s a huge improvement on the chaos that we had to put up with in bygone times, ORCID remains imperfect. It is not curated and, since it depends on each individual’s honesty and accuracy, is potentially open to error, fraud or abuse. Sooner or later I fear we shall be bounced by some scandal into a more rigorously policed system.

ORCID also provides a potential opportunity to solve another problem, namely the variability and inconstancy of researchers’ email addresses. These remain mostly tied to their institutions, so that when someone moves, or their university changes its name or its internet-based branding, or their mailbox irredeemably overflows with spam, their addresses can change abruptly and without anyone knowing. Mail addresses are also liable to be irretrievably deleted not only when a person’s contract of employment ends, but also when their supervisor forgets to click a box on a downloadable form, so as to confirm the renewal of their privileges for a further period. To avoid all this, why don’t we persuade the organization behind ORCID to issue unambiguous, for-ever mail addresses to every scientist in the world who registers, such as [16 digits]@orcid.org? It would also help editors check the credentials of authors, given that many of them nowadays resort to using email accounts with non-academic providers such as Microsoft or Google, to ensure continuity. If it catches on it might even spread to everyone in the world, since 16 digits suffices to create 10exp16 unambiguous IDs.

Another attribution issue arises with institution names. For enabling correspondence with authors, an email address is sufficient; exact postcodes, building names or grand-sounding laboratory titles have become irrelevant, at least for academic purposes. However, most institutions, as well as their funders and their governments, use the affiliations cited in the published literature for another purpose, namely to track the productivity of individuals, departments or whole institutions, often allocating resources and actual funding accordingly. Whether or not this is fair or justified, the frequent changes of institutional structure and nomenclature, right up to the name of entire universities, renders the whole process at best imprecise and in many cases a practical impossibility.

Such name changes are often bewildering even to those working in long-established institutions. Organizational changes are frequently a pointless exercise devised by an ambitious manager seeking to make their mark. Or a device for dismembering departments that are no longer fashionable, financially viable, or run by once powerful professors now long last their prime, and whom the central administration wants to cut down to size. Mergers, rebrandings and the creation of new subdivisions are commonly little more than a figleaf for, hence a signature of, failure.

Regardless of whatever diktat is issued from on high, many academics have only a rather approximate idea of what their site of employment is actually called or what is the current policy as to how its identity should be portrayed in publications. They prefer to focus their mental powers on science rather than on university admin rules, and may only be dimly aware of whether their department is now a division, a unit, a faculty, a school, a laboratory, an institute, a section or a nothing at all. Or what it was proposed to be changing to and whether the proposal was dropped, adopted, modified, shelved or remitted to an obscure committee. Or even the name of the discipline to which the department is supposedly devoted, and how it might differ from that of the chair which they hold, the degree that they teach or whatever it was when they joined it. They might not recall the name of its recent or historical benefactor and whether that identity is, was or ever will be part of its official name.

Publishers potentially contribute to the confusion and to putting mistaken affiliations into print. When submitting an article, we are usually provided with a drop-down menu, listing accepted institutional names. My own university often appears in such lists in several different guises and formulated in different languages. I have at times just given up trying to replace what is offered with what I consider to be correct. Even country names have to be selected from these menus, so that it is easy to click on ‘Fiji’ instead of ‘Finland’. Moreover, institutions—such as mine—that carry the status of post-towns with their own postcode, cause another layer of difficulty. I frequently have no other choice than to denote my affiliation as:

Institution: Tampere University

Address Line 1: Tampere University

City: Tampere University

Errors can usually be corrected during proofing, but authors are far more concerned to ensure that Figure 3A hasn’t been mistakenly presented in mirror-image, that the references have been correctly downloaded from a public database and renumbered in the right sequence, and that copy editors haven’t inverted the sense of a key interpretation of data. Misconstruction of the departmental name or address can easily be overlooked; or even introduced *after* proofing, by an over-zealous typesetter. In any case, all this is of importance only to a pedantic principal, dean, director, chairperson or minister of education. Or to a journalist or AI bot trying to compile one of those questionable league tables.

For the rest of us, who even cares about getting it right? In the end, does it matter in the slightest where laboratory work was conducted or whose name was on the plaque over the door? Nobody would want to know whether a particular flash of inspiration reported on page 6 was acquired during a visit to Cairo, in the showers at the gym, or after one too many at a cocktail party. So why bother giving the name of the building where the person sits when not working from home?

Personally I’d be quite happy with a one-word affiliation, just to alert readers to the fact that I am the Howy Jacobs in Tampere, not the one in Tahiti or Tajikistan. Or that I have finally moved on from Glasgow, Pasadena or wherever they thought I still was. The baffling profusion and misconstruction of affiliations that we live with is scientifically irrelevant. But, of course, we live in a world controlled by the bean-counters.

Finally, then, the correct attribution of published work to a specific cost centre is a bureaucratic task, not an academic one. So it should be the responsibility of the administrative services of our universities and institutes to identify the works that belong to their entity and score them accordingly, using whatever is their favoured metric. Leaving us scientists to do science.

### Supplementary information

Peer Review File

