# Supplementary material for: What’s in a @name?
Source: EMBO Rep. 2024 Jul 1;25(8):3167–8. doi: 10.1038/s44319-024-00194-7 (PMC11316044; doi:10.1038/s44319-024-00194-7)
Supplement: Supplementary file 1 — Peer Review File [file 44319_2024_194_MOESM1_ESM.pdf]

EMBO reports

What's in a @name?

Howard Jacobs

Corresponding author(s): Howard Jacobs (howard.jacobs@tuni.fi)

Review Timeline:

Submission Date:	19th Jun 24
Accepted:	19th Jun 24

Editor: Holger Breithaupt

Transaction Report: This article type is not peer reviewed.
